# Cyber-Biosecurity Risk Perceptions in the Biotech Sector

**DOI:** 10.3389/fbioe.2019.00136

**Published:** 2019-06-19

**Authors:** Kathryn Millett, Eduardo dos Santos, Piers D. Millett

**Affiliations:** Biosecure Ltd, Market Rasen, United Kingdom

**Keywords:** cyber-biosecurity, biotechnology, bioeconomy, infrastructure, risk perception, biosecurity, industry

## Abstract

The expanding digitization of the biological sciences places greater value on the data generated, information extrapolated and knowledge gained. Failing to protect data will affect a company or country's ability to position itself optimally in the forthcoming fourth industrial revolution. Further, more reliance on automation, distribution, and outsourcing in biotechnology makes its infrastructure a target. The equipment and service providers that drive physical research and development are also all connected online. Failing to protect these resources from intrusion increases the risk of accidental or deliberate harm, for example by the loss of control over biological products. Robust cybersecurity measures are therefore critical for both securing the data generated by the biotechnology sector as well as securing key infrastructure. Cyber-biosecurity is emerging multidisciplinary field that combines cybersecurity, biosecurity, and cyber-physical security as relates to biological systems (Murch et al., 2018). To better identify the perceived risks at the interface between cybersecurity and biosecurity, Biosecure conducted a pilot study that surveyed the opinions of a discrete set of international field leaders in biotechnology and cybersecurity. The survey was carried out online from October-November 2017. Key findings of the survey showed that cyber-biosecurity risks were considered to be difficult to characterize due to variations in types of threats, targets and potential impacts, and compounded by a notable variation between the level of sophistication or maturity of mitigation and response measures. Further research is therefore necessary bringing together the different communities focusing on these issues to develop a common language, better define the threats and discuss potential ways forward in addressing risks.

## Introduction

The development and recognition of “cyber-biosecurity” as an important element in securing data and products emerging from the biotechnology and biomedical sectors has predominantly emerged from the field of biosecurity. While the risks relating to accessing private biomedical data and the theft of valuable data from an intellectual property standpoint are well-known and recognized, the biosecurity implications of cyber intrusions relating to biotechnology infrastructure remain largely unknown in commercial biotechnology facilities.

To better gauge the current level of understanding and awareness of cyber-biosecurity risks in the biotechnology sector and identify how the risks are perceived, Biosecure conducted a pilot survey targeting a discrete set of international leaders in the fields of biotechnology and cybersecurity.

## Methodology

To conduct a discrete pilot survey of the types and level of cyber-biosecurity risks identified in the field of biotechnology, a short questionnaire comprising 12 questions that was posted securely online. The questions posed were a mix of multiple choice and open-ended questions, divided across the themes of risk perception and awareness, risk mitigation capacities and resources, and the urgency of, and potential avenues for, any future action. The questions were reviewed by an expert in qualitative methodology to eliminate any issues of bias.

The survey described in this paper was conducted in accordance with the Declaration of Helsinki and all participants provided informed consent in writing (World Medical Association, 2013). The survey described is not considered research by the UK National Health Service and Medical Research Council and does not require review by a Research Ethics Committee. In addition, Biosecure Ltd. funded the survey using its own corporate funds. Biosecure Ltd. does not, and has not, received US Federal research funding. As a result, the survey described in this paper was performed in accordance with relevant institutional and national guidelines.

Twenty-six individuals were invited to participate from across the biotech and cybersecurity sector. Invitees from the biotech sector included founders of small to medium biotechnology companies in the United States and United Kingdom, senior management of large biotechnology companies (with an international footprint), representatives of industry, venture capitalists specializing in biotechnology, and advisors to the above on security issues. The individuals approached in the cybersecurity sector included industry specialists, leading academics, national government experts, experts in leading think tanks, and specialists within intergovernmental organizations.

Overall, of the 26 invited questionnaire participants, 13 agreed to participate. The responses were anonymized.

## Survey Results

The results of the survey were assessed according to four key areas: (1) assessing the threat; (2) assessing threat mitigation and response capacity; (3) available tools and resources; and, (4) recommended next steps. The key findings under each of these areas are elaborated below and summarized in Table 1.

**Table 1:** Relative risk perception of different cybersecurity threats to biotech.

	**No or minimal risk**	**Risk comparable to normal operating standards**	**Elevated or severe risk**
An incident in which an unauthorized actor takes control of infrastructure (e.g., lab equipment, lab control systems, or even a fully automated robot lab)	2	2	9
An incident in which an unauthorized actor accesses data, information, or knowledge that is not in the public domain	0	2	11
An incident in which an unauthorized actor is able to circumvent security controls, such as those used to screen orders and customers amongst certain biotech service providers	0	3	9
An incident in which an unauthorized actor is able to secretly change data, information, or knowledge	0	1	12
An incident in which an unauthorized actor is able to interrupt the functioning of lab systems	0	4	9
An incident originating from a compromise in the supply chain	0	2	9

*White, No response; Yellow, 1 to 5 responses; Orange, 6 to 10 responses; Red, Over 10 responses*.

### Assessing the Threat

Over two-thirds of respondents deemed the risks posed to the biotechnology sector by cyber threats and intrusions as elevated or severe when compared to normal operating standards in the biotech industry. The two scenarios perceived to pose the greatest risk were: unauthorized access to data, information, or knowledge outside the public domain; and unauthorized actors able to secretly change data, information, or knowledge. In only one scenario (in which an unauthorized actor takes control of infrastructure) did any respondent think there was no or minimal risk.

- When asked to identify different types of risks from cybersecurity breaches in the biotech sector, participants noted potential negative impacts from:
- The theft, elimination or ransom of data, algorithms, or software with a direct or indirect impact on R&D or commercial operations;
- Modification of data, algorithms, or software with a direct or indirect impact on research and development or commercial operations;
- The loss of intellectual property or commercial advantage by data, algorithms, or software being available to competitors;
- Potential for the disabling or disruption of important systems or infrastructure leading to disruption of commercial operations or impeding good manufacturing practices;
- Manipulation of bio-manufacturing or automated systems to create risks.

Respondents ranked states and proxies used by states as the type of actor posing the greatest risk, with lone individuals viewed as generating the least risk. This survey did not differentiate between insider or outsider threats, regardless of whether states, groups or lone individuals. This may be an area ripe for further study.

All participants considered that cyber-biosecurity risks posed a real and current threat, but that these were not, or only partially, being addressed within the biotech sector. In part, this was considered due to a lack of awareness and information within the biotech community, with one participant noting that “[M]any companies are unaware of the intensity of outsider threats because they are not actively monitoring these activities.”

### Assessing Current Threat Mitigation and Response Capacities

While noting the lack of sufficient information on the type and level of biorisks to the biotech sector by cyber intrusions, over seventy-five per cent (75%) of participants indicated that their organizations had undertaken some efforts to address cybersecurity issues, and ninety per cent (90%) of these reported that such measures were regularly reviewed.

However, the comprehensiveness and maturity of mitigation efforts were reported as being varied, with some participants reporting that their efforts were only in the nascent stages. One respondent, for example, noted that their activities had been “…mostly discussions that it will be a problem but they have no idea nor urge to address it.” Another noted that the issues had been considered “[F]airly deeply, although [we] have not… done any work to implement anything.”

By contrast, other participants had begun integrating cybersecurity into their business with a participant reporting that “[W]e have considered security implications in our technology development at all levels… partner technologies we integrate have always required a careful discussion of the security implications that flow from their use, and as a result we rely heavily on technologies from vendors such as Google and Microsoft that have strong security cultures.”

In addition to variances in awareness and the perceived risks posed by cyber-attacks to biological facilities and equipment, respondents pinpointed the lack of available resources as a limiting factor for addressing cyber-biosecurity. Over ninety per cent (90%) of participants expressed a strong view that insufficient time and resources are being dedicated to dealing with these risks. One participant noted they “have not yet had the resources to do formal red team testing of our systems” and another commented that “[S]ufficient time and resources are almost never dedicated to dealing with risks from cybersecurity; biotech is no exception.” Further, it was remarked that “[D]ealing with cybersecurity breaches is not a one size fits all process. Filling the gaps on the topic requires a tailored approach for each company, entity, or facility. By performing a comprehensive gap analysis for each entity, the answer to this question can be discovered.”

**Figure 1:**
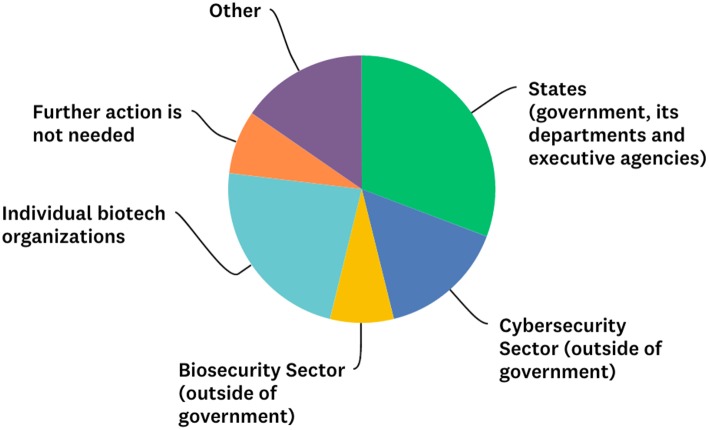
Views as to the appropriate primary actor in addressing any risks from cybersecurity breaches in biotech.

When asked their view on the appropriate agency to take the lead in addressing any risks from cybersecurity breaches in biotechnology, participants showed a wide divergence of opinion (Figure 1) suggesting that a multi-stakeholder approach may be warranted.

### Available Tools and Resources

Over seventy-five (75%) of respondents were unaware of any dedicated resources (reports, guidance, standards, etc.) for dealing with risks from cybersecurity breaches in biotechnology. Those that were aware of existing resources highlighted internal company resources, broader standards that incorporated aspects of biosecurity and cybersecurity but which did not specifically address the overlap, or country-specific resources, such as National Institute of Standards and Technology and FBI outreach agents in the USA.

However, there was greater awareness (50%) of the existence of “dedicated support for dealing with this issue (such as hotlines, reporting infrastructure, national experts, commercial services, etc.” with two thirds of those respondents aware of support citing the Weapons of Mass Destruction (WMD) Directorate of the FBI and one respondent citing private company, Ebiosec. No participant identified sources of support that specifically address the cybersecurity needs of the biotech sector outside of the USA.

### Recommended Next Steps in Addressing Cyber-Biosecurity

Several respondents pointed to efforts to address gaps in the interface between cyber- and biosecurity including sponsored meetings and, in a few cases, having specifically allocated staff time to addressing these issues. In addition, notice has been made of the emergence of new actors in the field, including such as companies like Ebiosec which provides services to “manage, model, secure, and visualize their data-driven life sciences operations^1^.” The founders of this company also manage an online portal for “fostering discussions and sharing information, events and tools to secure the digital dimension of the biothreat^2^.”

However, the majority of participants acknowledged that much more needs to be done to bring together the communities addressing biosecurity and cybersecurity, and identify effective measures and approaches to mitigate and prevent the risks, including fine tuning broader regulatory approaches to help foster a cybersecurity culture. One participant noted “Biotech does not think about security other than more traditional biosecurity and biosafety; security communities do not understand biotech (focused on traditional telecoms and digital).”

A number of issues warranting increased attention were also identified, including: the implications of new supply/value chains; techno-espionage or potential for business model/regulatory disruptions; loss of public/political trust resulting from inactivity; and how cybersecurity risk impacts competitiveness of biotechnology companies.

## Conclusion

The issue of cyber-biosecurity is not well-known or understood, even among biotechnology and cybersecurity experts. A concerted effort to develop this emerging field, define, and foster awareness of the threats and craft a common language is therefore a pressing need as the digital age of biology progresses.

Opportunities are needed to bring together communities focusing on these issues, and begin work on areas of common interest and the means to address the identified risks. Strengthened multi-stakeholder capacity is needed to work at the interface between cybersecurity and biosecurity, and support and resources should be invested in further understanding cybersecurity risks in the biotechnology sector in order to develop appropriate counter measures.

## Author Contributions

KM is the lead author of this paper. PM and KM devised and carried out the survey. EdS provided technical assistance during the survey and conducted a literature review on cyberbiosecurity.

### Conflict of Interest Statement

KM and PM are founders and owners of Biosecure Ltd. Biosecure Ltd. does not, and has not, received US Federal research funding. The remaining author declares that the research was conducted in the absence of any commercial or financial relationships that could be construed as a potential conflict of interest. The reviewer GK declared a past collaboration with one of the authors, PM, to the handling Editor.

## Footnotes

^1^See http://ebiosec.com/

^2^See http://information-biosecurity.org/
